# Comparison of clinical efficacy of robot-assisted and freehand core decompression in the treatment of osteonecrosis of the femoral head: a systematic review and meta-analysis

**DOI:** 10.1186/s12891-024-07592-x

**Published:** 2024-06-18

**Authors:** Wensi Ouyang, Guimei Guo, Tianpei Jiang, Changwei Zhao, Xiaoling Zhou

**Affiliations:** 1 Changchun University of Chinese Medicine, Changchun, 130117 China; 2 Hospital Affiliated to Changchun University of Traditional Chinese Medicine, Changchun, 130021 China

**Keywords:** Robotics, Core decompression, Osteonecrosis of the femoral head, Meta-analysis, Systematic review

## Abstract

**Objective:**

At present, the core decompression (CD) has become the main surgical procedure for the treatment of osteonecrosis of the femoral head (ONFH); however, the CD surgery requires high operator experience and repeated fluoroscopy increases the radiation damage to patients, and medical staff. This article compares the clinical efficacy of robot-assisted and freehand CD for ONFH by meta-analysis.

**Methods:**

Computer searches of PubMed, Web of Science, Embase, Cochrane Library, Chinese National Knowledge Infrastructure, China Science and Technology Journal Database, WanFang, and Chinese BioMedical Literature Database were conducted from the time of database inception to November 15, 2023. The literature on the clinical efficacy of robot-assisted and freehand CD in the treatment of ONFH was collected. Two researchers independently screened the literature according to the inclusion and exclusion criteria, extracted data, and strictly evaluated the quality of the included literature. Outcome measures encompassed operative duration, intraoperative blood loss volume, frequency of intraoperative fluoroscopies, visual analog scale (VAS) score, Harris hip score (HHS), complications, and radiographic progression. Data synthesis was carried out using Review Manager 5.4.1 software. The quality of evidence was evaluated according to Grades of Recommendation Assessment Development and Evaluation (GRADE) standards.

**Results:**

Seven retrospective cohort studies involving 355 patients were included in the study. The results of meta-analysis showed that in the robot-assisted group, the operative duration (MD = -17.60, 95% CI: -23.41 to -11.78, *P* < 0.001), intraoperative blood loss volume (MD = -19.98, 95% CI: -28.84 to -11.11, *P* < 0.001), frequency of intraoperative fluoroscopies (MD = -6.60, 95% CI: -9.01 to -4.20, *P* < 0.001), and ΔVAS score (MD = -0.45, 95% CI: -0.67 to -0.22, *P* < 0.001) were significantly better than those in the freehand group. The GRADE evidence evaluation showed **Δ**VAS score as low quality and other indicators as very low quality. There was no significant difference in the terms of ΔHHS (MD = 0.51, 95% CI: -1.34 to 2.35, *P* = 0.59), complications (RR = 0.30, 95% CI: 0.03 to 2.74, *P* = 0.29), and radiographic progression (RR = 0.50, 95% CI: 0.25 to 1.02, *P* = 0.06) between the two groups.

**Conclusion:**

There is limited evidence showing the benefit of robot-assisted therapy for treatment of ONFH patients, and much of it is of low quality. Therefore, caution should be exercised in interpreting these results. It is recommended that more high-quality studies be conducted to validate these findings in future studies.

**Systematic review registration:**

https://www.crd.york.ac.uk/prospero/ #recordDetails, CRD42023420593.

**Supplementary Information:**

The online version contains supplementary material available at 10.1186/s12891-024-07592-x.

## Introduction

Osteonecrosis of the femoral head (ONFH) represents a significant orthopedic enigma, as its exact pathogenesis remains elusive, making its treatment a considerable challenge for orthopedic surgeons [1–2]. Alarming data suggest that without timely intervention, over 90% of ONFH cases will culminate in the collapse of the femoral head within five years [3–4]. Consequently, when many patients seek their initial consultation, the integrity of the hip joint is already compromised to the point where hip replacement becomes inevitable [5–6]. This progression places a considerable strain on patients and imposes a profound socioeconomic burden [7]. Therefore, the imperative lies in devising strategies to preemptively address ONFH before the onset of femoral head collapse, thereby decelerating the disease’s trajectory and postponing the need for joint replacement.

Core decompression (CD) has established itself as a pivotal therapeutic strategy in the clinical management of ONFH [8–9]. Traditional CD relies heavily on the expertise of the surgeon, aided by multiple fluoroscopic imaging under X-ray, to meticulously target and decompress the necrotic zone. Nevertheless, this method is not devoid of risks. There is the ever-present danger of inadvertently puncturing the cartilage or damaging blood vessels and neuromuscular structures during the procedure [10–11]. The advent and assimilation of robotic navigation technology into clinical orthopedics introduces a promising alternative, touting advantages such as enhanced precision, reduced reliance on fluoroscopy, and bolstered patient safety [12–13]. However, the development of robot-assisted technology is still in its infancy, and its clinical efficacy and safety are still uncertain. Hence, this meta-analysis aimed to determine whether the robot-assisted CD technique offers an advantage in clinical efficacy compared with the X-ray freehand CD technique.

## Methods and materials

### Protocol registration

This systematic review and meta-analysis was structured in adherence to the guidelines of the Cochrane Handbook for Systematic Reviews and was reported as per the Preferred Reporting Items for Systematic Reviews and Meta-Analyses [14]. The meta-analysis was registered on the PROSPERO platform under the identifier CRD42023420593.

### Search strategy

Collaboratively, two authors (W.S.O.Y. and G.M.G.) embarked on a comprehensive exploration of four English electronic databases (PubMed, Web of Science, Embase, and Cochrane Library) and four Chinese databases (Chinese National Knowledge Infrastructure, China Science and Technology Journal Database, WanFang, and Chinese BioMedical Literature Database) covering their inception to November 15, 2023. Search terms embraced “robotic”, “robot positioning”, “navigated”, “osteonecrosis of the femoral head”, “femur head necrosis”, “ONFH”, and “FHN”. The search strategy amalgamated theme-based terms with free-text words customized to each database’s specifications. Additionally, to ensure a comprehensive search, references from the included articles were scanned for any other potentially relevant studies. Only articles in English and Chinese were included. The specific PubMed search algorithm was: (((robotic [Title/Abstract]) OR (robot positioning [Title/Abstract]) OR (navigated [Title/Abstract]) AND (osteonecrosis of the femoral head [Mesh]) OR ((femur head necrosis [Title/Abstract])) OR (ONFH [Title/Abstract])) OR (FHN [Title/Abstract]). A thorough breakdown of the search methodologies employed is available in Supplementary Material 1.

#### Eligibility criteria

1. Research Types: Published randomized controlled trial studies and retrospective cohort studies.
2. Participants: Inclusion involved patients diagnosed with ONFH based on recognized criteria. Staging references were the Association Research Circulation Osseous stages, Ficat stages, and Steinberg stages [15–17]. No restrictions were imposed based on demographic factors such as age, gender, ethnicity, geographical location, or the study’s origin.
3. Interventions: Control group: CD therapy by freehand under X-ray fluoroscopy. Intervention group: CD therapy assisted by robotic positioning system.
4. Type of outcome measures: Included studies were required to include one of the following outcomes: operative duration, intraoperative blood loss volume, frequency of intraoperative fluoroscopies, visual analog scale (VAS) score, Harris hip score (HHS), complications, and radiographic progression.

#### Exclusion criteria

1. Literature with overlapping data or duplicate publications.
2. Literature reviews, case reports, animal experiments, basic experimental studies, letters, and review articles.
3. Primary or relevant outcome indicators are unavailable.

### Data extraction

Titles and abstracts of the gathered studies were meticulously scrutinized by two independent reviewers (W.S.O.Y. and T.P.J.). A thorough reading of the full-text articles ensued to determine alignment with both inclusion and exclusion criteria. Any discordance was resolved through in-depth discussions and, if necessary, in collaboration with a third reviewer (X.L.Z.) to reach a unanimous decision. Two authors (W.S.O.Y. and G.M.G.) employed a systematic data extraction template to mine essential study features, encompassing title, publication year, lead author, country, study design, sample size, treatment strategies, treatment duration, primary outcome measures, and follow-up time. Key outcomes were extracted separately by two other reviewers (C.W.Z. and X.L.Z.) for data synthesis. A collaborative consensus approach was adopted in data extraction discrepancies between reviewers, involving all reviewers.

### Literature quality assessment

The methodological quality of each included randomized controlled trial study was separately assessed by two review authors (W.S.O.Y. and G.M.G.) by using the Cochrane Risk of Bias tool [18]. All included retrospective studies underwent a rigorous quality assessment utilizing the Newcastle-Ottawa Scale (NOS). This scale evaluates pivotal aspects such as the selection of study populations, the comparison of study cohorts, and the methods employed for outcome assessments [19]. If the two researchers disagree, it can be resolved by consulting a third reviewer author (C.W.Z.).

### Quality of evidence assessment

We used the principles of the Grades of Recommendation Assessment Development and Evaluation (GRADE) system to assess the quality of the body of evidence associated with outcomes [20]. Developed to grade the overall certainty of a body of evidence, this approach incorporates six main domains: limitation in study design, risk of bias, inconsistency of results, indirectness, imprecision, and publication bias [21]. Two review authors (W.S.O.Y. and G.M.G.) separately assessed each domain for each selected outcome. If the two researchers disagree, it can be resolved by consulting a third reviewer author (C.W.Z.). We documented all decisions regarding up- or down-grading the certainty of evidence to ensure transparency.

### Statistical analysis

Statistical computations were executed using the Review Manager 5.4.1 software (provided by the Cochrane Collaboration, Oxford, UK). The Risk Ratio (RR) was employed for binary data, while the Mean Difference (MD) served for continuous data types. Both metrics were accompanied by a 95% Confidence Interval (CI) to encapsulate the effect magnitude when juxtaposing treatment and control groups. Inherent heterogeneity was assessed using the χ^2^ test and quantified by the I^2^ value. An I^2^ value of ≤ 50% coupled with *P* ≥ 0.1 suggested homogeneity across studies, warranting the application of a fixed-effects model. Conversely, an I^2^ value surpassing 50% alongside *P* ≤ 0.1 indicated significant heterogeneity, prompting the utilization of a random-effects model.

## Results

### Search results

**Fig. 1:**
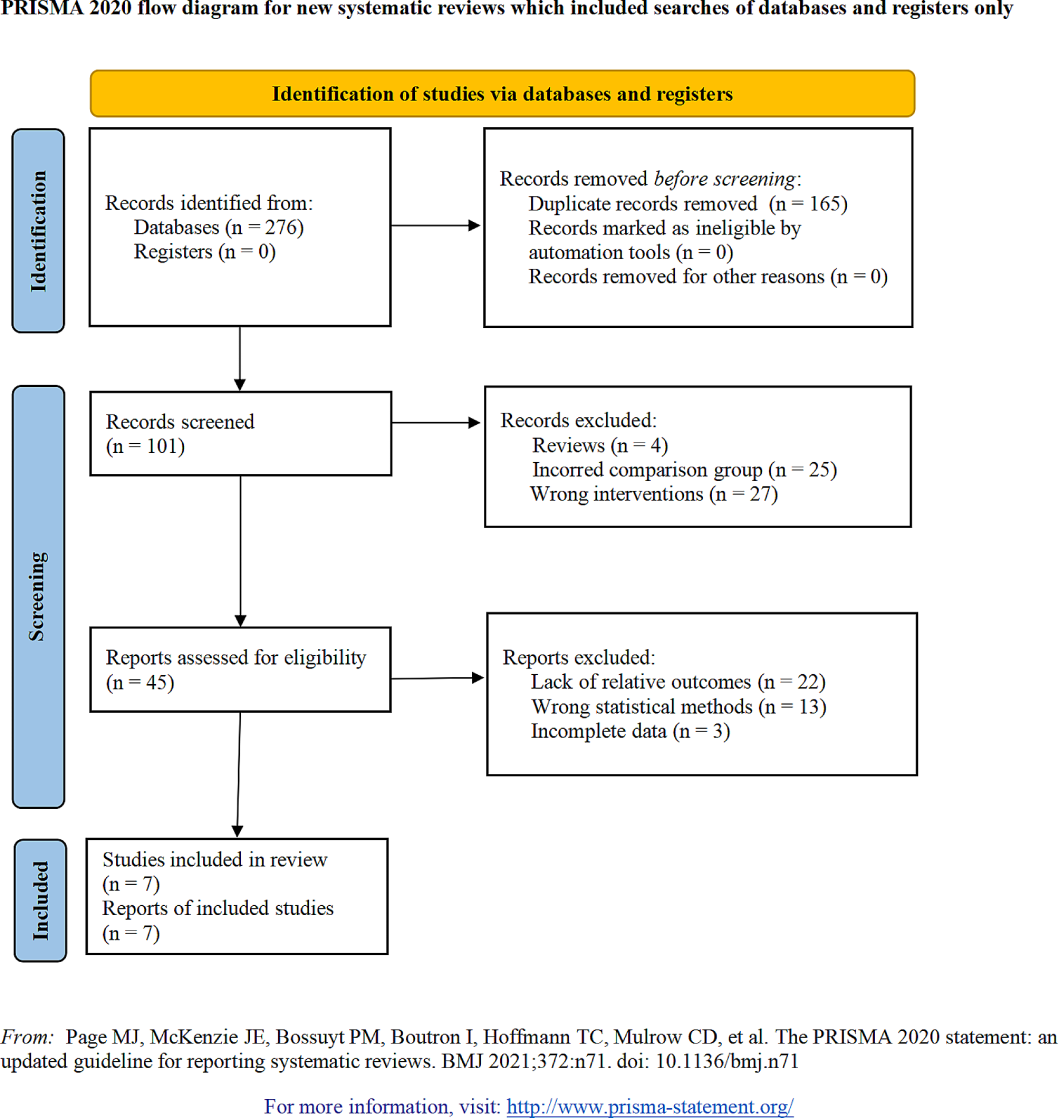
Literature screening process and results

Out of an initial pool of 276 potential articles concerning robot-assisted CD treatment for ONFH, 165 duplicates were identified and removed. Subsequent screenings based on titles and abstracts eliminated another 56 articles, while 38 more were excluded following full-text reviews and criteria assessment. Consequently, this meta-analysis incorporated seven articles [22–28] (Fig. 1).

### Study characteristics

In the selected studies, 355 adult ONFH patients were documented. Among them, 183 were in the freehand group, and 172 were in the robot-assisted group. Every included study specified clear criteria for inclusion and exclusion, and consistent baseline data was observed across the freehand and robot-assisted cohorts (Table 1). Regarding financial support, four out of the seven studies [23–26] were funded by governmental bodies or professional entities, whereas the remaining 3 [22, 27–28] did not specify their funding source.

**Table 1:** Basic characteristics of the seven studies included in the meta-analysis

Authors & Country	Study design	Sample (M/F)	Hip (M/F)	Age (years)	Diagnostic standard	Disease stage	Follow-up (months)
Tian et al., 2023 [22] China	Retrospective cohort study	T: 17 (NA) C: 24 (NA)	T: 28 (NA) C: 42 (NA)	T: 40.8 ± 8.1 C: 42.1 ± 6.4	ARCO	II	14.6 ± 4.8
Liu et al., 2022 [23] China	Retrospective cohort study	T: 18 (13/5) C: 13 (9/4)	T: NA C: NA	T: 36.8 ± 4.8 C: 34.5 ± 6.1	Steinberg	II/III/IV	6
Zhang et al., 2022 [24] China	Retrospective case control study	T: 30 (19/11) C: 30 (20/10)	T: NA C: NA	T: 38.50 ± 10.61 C: 40.63 ± 10.63	ARCO	I	T: 23.40 ± 1.65 C: 23.30 ± 1.66
Li et al., 2022 [25] China	Retrospective cohort study	T: 50 (36/14) C: 50 (37/13)	T: NA C: NA	T: 47.52 ± 12.50 C: 50.68 ± 13.42	ARCO	II	28.53 ± 0.5
Luo et al., 2020 [26] China	Retrospective cohort study	T: 18 (12/6) C: 22 (12/10)	T: 26 (NA) C: 29 (NA)	T: 50.91 ± 7.59 C: 51.39 ± 7.38	Ficat	I/II	6
Luo J et al., 2020 [27] China	Retrospective case control study	T: 30 (18/12) C: 33 (20/13)	T: 41 (NA) C: 46 (NA)	T: 53.00 ± 7.09 C: 50.00 ± 8.84	Ficat	I/II	6
Bi et al., 2019 [28] China	Retrospective case control study	T: 9 (7/2) C: 11 (8/3)	T: 16 (NA) C: 20 (NA)	T: 34.88 ± 3.81 C: 35.30 ± 4.72	ARCO	II	T: 26.8 ± 3.42 C: 26.4 ± 3.65

**Note**: ARCO, Association Research Circulation Osseous; C, Control group; NA, Not available; T, Treatment group

### Demographic and staging

A total of 355 patients were included in the systematic review and meta-analysis. The age of the patients enrolled in these included studies ranging from 34.5 to 53.0. The included studies had a minimum follow-up of 6 months. The study of the longest follow-up came from China, where Li’s study [25] reported a 28-year result. Three kinds of diagnostic standards were utilized in all of the studies. Among them, Four studies [22, 24–25, 28] followed ARCO classification, 2 studies [26–27] reported data on the basis of Ficat classification, and the left 1 study [23] used the steinberg staging system.

### Bias risk assessment

Included seven literature articles [22–28] were all retrospective cohort studies, with no randomized controlled studies. According to the NOS assessment, there were four literature [23–25, 28] scores of eight and the remaining three literature [22, 26–27] scores of seven (additional data in Supplementary Material 1: Table S1).

### Meta-analysis outcomes

#### Operative duration

**Fig. 2:**
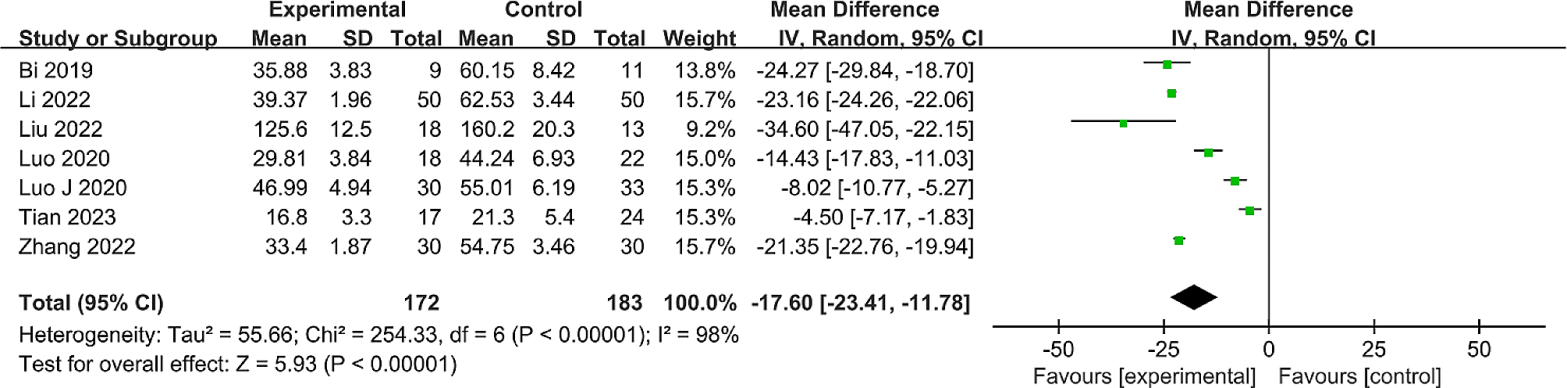
Forest plot of the meta-analysis comparing the operative duration

All seven studies [22–28] comprising 355 patients compared operative duration across both groups. Significant heterogeneity was observed (*P* < 0.00001, I^2^ = 98%), necessitating the application of a random effects model. The results signified that the operative duration was statistically shorter in the robot-assisted group (MD = -17.60, 95% CI: -23.41 to -11.78, *P* < 0.001) (Fig. 2). Sensitivity analysis was then conducted by individually excluding each study. The outcome remained consistent, reinforcing the stability of the meta-analysis results (additional data in Supplementary Material 1: Table S2).

#### Intraoperative blood loss volume

**Fig. 3:**
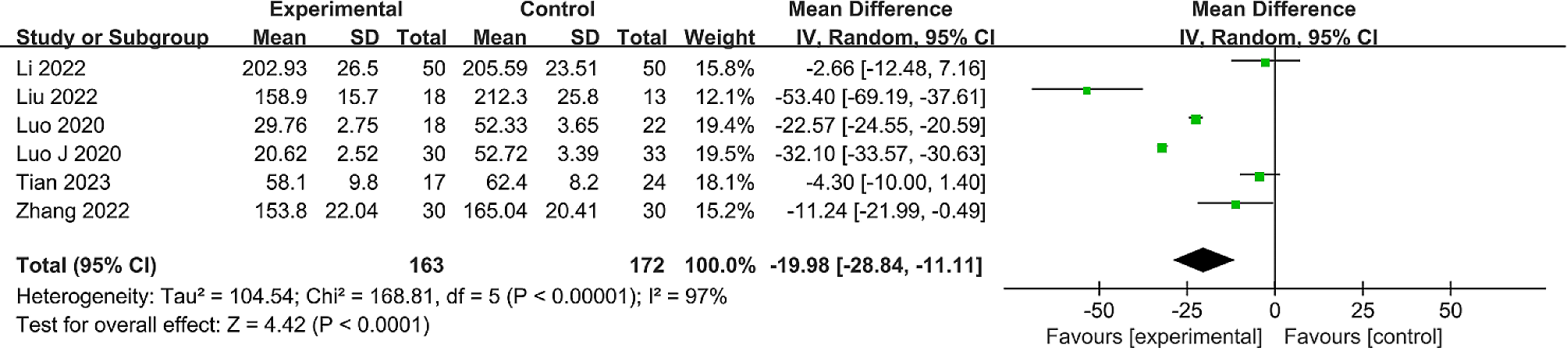
Forest plot of the meta-analysis comparing the intraoperative blood loss volume

Out of the studies, 6 [22–27], with a collective patient count of 335, provided data regarding intraoperative blood loss volume. A notable heterogeneity was recorded (*P* < 0.00001, I^2^ = 97%), warranting the use of a random effects model. The data revealed a statistically significant reduction in intraoperative blood loss volume for the robot-assisted group (MD = -19.98, 95% CI: -28.84 to -11.11, *P* < 0.001) (Fig. 3). A sensitivity analysis, excluding individual studies, was executed, which reasserted the stability of the derived results from the meta-analysis (additional details in Supplementary Material 1: Table S3).

#### Frequency of intraoperative fluoroscopies

**Fig. 4:**
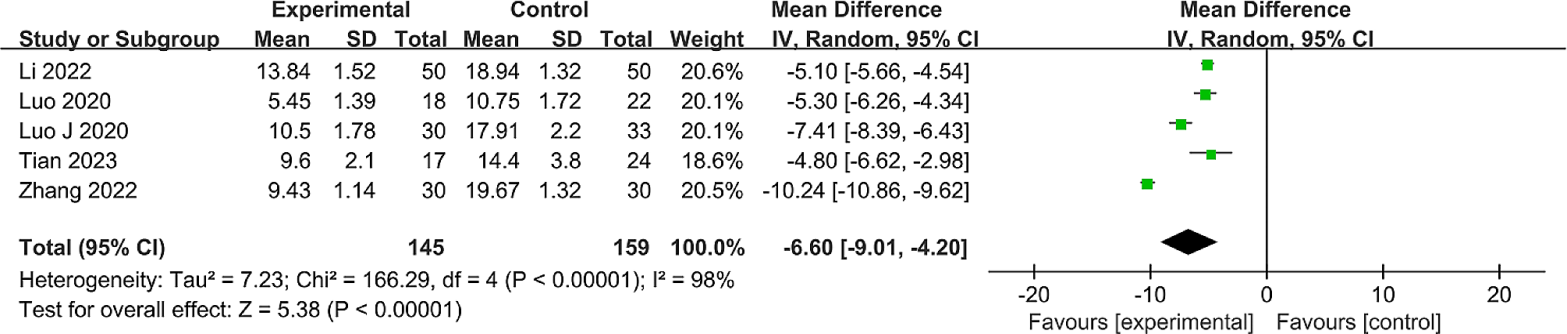
Forest plot of the meta-analysis comparing the frequency of intraoperative fluoroscopies

The frequency of intraoperative fluoroscopies between the groups was reported from the five studies [22, 24–27] of 304 patients. Significant heterogeneity was observed (*P* < 0.00001, I^2^ = 98%), prompting the utilization of a random effects model. The analysis indicated a statistically significant reduction in the frequency of intraoperative fluoroscopies for the robot-assisted group (MD = -6.60, 95% CI: -9.01 to -4.20, *P* < 0.001) (Fig. 4). A sensitivity analysis, where each study was individually excluded, confirmed the stability of the meta-analysis results (additional details in Supplementary Material 1: Table S4).

#### ΔVAS score

**Fig. 5:**
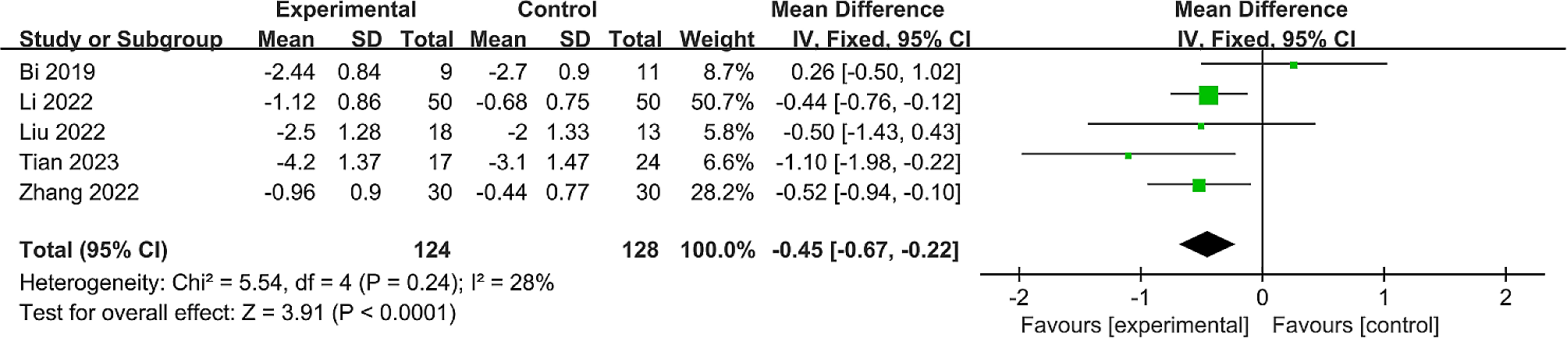
Forest plot of the meta-analysis comparing the ΔVAS score

Out of five studies [22–25, 28] that included 252 participants, the change in VAS score was reported from baseline to the last follow-up. Minimal heterogeneity was found (*P* = 0.24, I^2^ = 28%), allowing the use of a fixed effect model. The analysis highlighted a significant difference in ΔVAS score, favoring the robot-assisted group (MD = -0.45, 95% CI: -0.67 to -0.22, *P* < 0.001) (Fig. 5).

#### ΔHHS

**Fig. 6:**
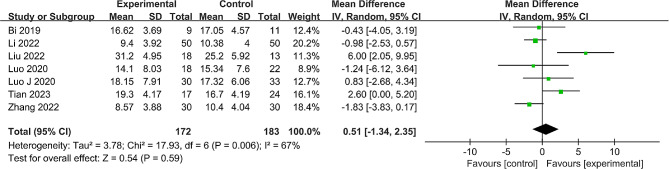
Forest plot of the meta-analysis comparing the ΔHHS

Seven studies [22–28], comprising 355 participants, detailed the changes in the HHS from baseline to the last follow-up. Notable heterogeneity was observed (*P* = 0.006, I^2^ = 67%), necessitating a random effects model. Interestingly, no significant difference in ΔHHS was detected (MD = 0.51, 95% CI: -1.34 to 2.35, *P* = 0.59) (Fig. 6). To further analyze the stability of the results, we made sensitivity analyses and excluded each study from the analysis. Excluding other studies, the meta-analysis result indicated that the data analysis was stable. After excluding Liu’s study [23], the heterogeneity changed significantly (*P* = 0.15, I^2^ = 39%). This suggested that this study may have been the source of heterogeneity in ΔHHS data (additional details in Supplementary Material 1: Table S5).

#### Complications

**Fig. 7:**
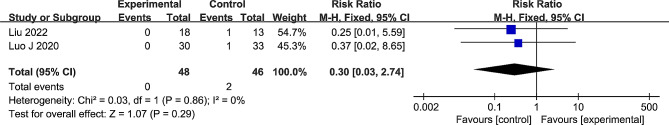
Forest plot of the meta-analysis comparing the complications

Only two studies [23, 27] with 94 participants reported complications between the groups. In Liu’s study [23], one instance of deep vein thrombosis in the freehand group was noted, while Luo J’s study [27] documented a single case of wound infection, also in the freehand group. A fixed effect model was utilized given the low heterogeneity (*P* = 0.86, I^2^ = 0%). The results did not show any statistical significance concerning complications (RR = 0.30, 95% CI: 0.03 to 2.74, *P* = 0.29) (Fig. 7).

#### Radiographic progression

**Fig. 8:**
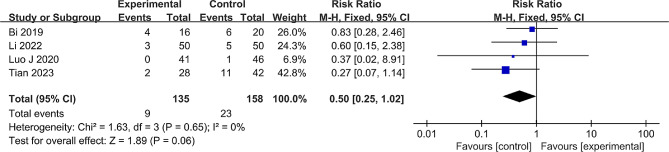
Forest plot of the meta-analysis comparing the radiographic progression

Four studies [22, 25, 27–28] that included 293 hips reported radiographic progression between the groups. Minimal heterogeneity was found (*P* = 0.65, I^2^ = 0%), allowing the use of a fixed effect model. The results did not show any statistical significance concerning radiographic progression (RR = 0.50, 95% CI: 0.25 to 1.02, *P* = 0.06) (Fig. 8).

### GRADE evaluation

Based on the principles of the GRADE evaluation, we evaluated the quality of the evidence provided via the operative duration, intraoperative blood loss volume, frequency of intraoperative fluoroscopies, and **Δ**VAS score. Table S6 shows that, except for **Δ**VAS score which was classified as low in quality, the others were evaluated as very low quality.

## Discussion

### Summary of evidence

In this analysis of seven retrospective studies with a collective of 355 patients, we aimed to discern the efficacy and safety of robot-assisted CD in the management of ONFH. Our findings reveal that the robot-assisted group offers a superior approach over the freehand group in various aspects such as operative duration, intraoperative blood loss volume, frequency of intraoperative fluoroscopies, and ΔVAS score; the differences were statistically significant. However, no meaningful differences emerged between the two groups when evaluating parameters like ΔHHS, complications, and radiographic progression.

### Analysis of findings

One salient benefit of employing robot-assisted CD for ONFH treatment is the observed reduction in operative duration. This is attributed to robot-assisted techniques that utilize advanced digital technology which harnesses marker points on the robotic arm, preoperative CT reconstruction data, and intraoperative X-ray fluoroscopic images. Combining these resources empowers surgeons to meticulously plan their surgical pathway, ascertain the optimal nail entry point with a single guide needle measurement, and perform all surgical tasks via a minimally invasive incision. Not only does this significantly cut down the operative duration [22, 25], but it also diminishes the patient’s intraoperative incision exposure time, consequently lowering the risk of joint infections. However, we must not overlook the observed heterogeneity. Potential causes include the relatively nascent adoption of robot-assisted CD by specific research centers, the consequent surgical inexperience, and differing calculations of surgery time among centers. Some research facilities consider the operative duration from skin disinfection, which typically necessitates an extended preoperative preparation [25, 27], while others [28] start the clock at the skin incision. Such inconsistencies can inadvertently skew the research outcomes.

Another pivotal metric for gauging surgical safety and quality is the intraoperative blood loss volume. Excessive intraoperative bleeding during femoral head surgery elevates the risks of perioperative complications, prolongs hospital stay, and subsequently hikes up hospitalization expenses. As highlighted by Liu’s study [23], orthopedic robotic systems stand out for their precision. These robotic aids enable surgeons to meticulously plan their paths and pinpoint targets accurately, minimizing damage to the blood vessels and surrounding tissues during surgery.

Our analysis underscores the advantages of robot-assisted groups in managing ONFH. Evident benefits include shortened operative duration, reduced intraoperative blood loss volume, and a significant reduction in the frequency of intraoperative fluoroscopies. Considering the often-underplayed radiation risks in orthopedic surgery, the latter is especially crucial. Freehand group necessitates multiple uses of C-arm fluoroscopy to achieve desired results, inadvertently increasing radiation exposure for both healthcare professionals and patients. With accumulating radiation exposure, risks such as compromised immunity, heightened susceptibility to blood disorders, and cancer become paramount concerns [29–31]. Tian’s study [22] suggested that robot-assisted techniques can enable intraoperative femoral drilling in a three-dimensional view by matching preoperative three-dimensional reconstructed images with intraoperative X-ray images. Similarly, Luo’s study [26] indicated that the intraoperative use of robot-assisted techniques can reduce the frequency of intraoperative fluoroscopies, which has some clinical application prospects.

For early-stage ONFH patients, the typical manifestation is hip pain, making the VAS score a pertinent gauge for patient outcomes. Simultaneously, the HHS remains a benchmark for postoperative hip function assessment. Our comparative analysis, grounded in statistical evaluation of the difference between preoperative and subsequent follow-up VAS score and HHS [32], indicates that robot-assisted CD’s minimally invasive nature, which involves smaller incisions and minimal collateral tissue damage, can effectively attenuate postoperative pain, as evidenced in Luo J’s study [27]. The lack of discernable difference in ΔHHS might be attributed to its inherently subjective nature. Patient perceptions and experiences can introduce biases, suggesting the need for more extensive, rigorous studies to validate these findings. In theory, robotic surgery can reduce the number of medical injuries by positioning the procedure more precisely. While robot-assisted CD emerges as a promising advancement in ONFH management, further rigorous studies are imperative for a more comprehensive understanding and potential wider clinical adoption.

For the patient, delaying the collapse of the femoral head is the primary principle in the treatment of ONFH. Tian’s study indicated that robot-assisted therapy could improve the success rate of core decompression and effectively reduce the collapse rate of early- and mid-stage ONFH [22]. Meanwhile, in the final follow-up observation, it was found that most of the patients in the robot-assisted therapy group had intact femoral head morphology. Li’s study concluded that robot-assisted techniques are effective in improving surgical accuracy and minimizing damage to cortical bone [25]. Even when judgments about radiographic progression were made, there was considerable subjectivity. This also emphasizes the importance of intervention concealment and implementation of blinding.

The quality of the included studies was evaluated using the GRADE system, which adopts a highly structured approach to classify the level of evidence and clearly presents the evaluation items in itemized lists so that clinicians can understand the effectiveness and feasibility of the treatment measures on their own and then make clinical decisions. Among the metrics in this study, the **Δ**VAS score was rated as low quality, the operative duration, intraoperative blood loss volume, and frequency of intraoperative fluoroscopies were rated as very low quality. The reasons may be related to the fact that the included studies were all non-randomized controlled trials, large heterogeneity among some studies, and publication bias. Hence, there is still a need to include higher quality studies to improve the level of evidence in the future. In the clinical management of patients with ONFH, there is still a need for a comprehensive assessment of the patient’s overall condition in order to make scientific clinical decisions.

### Limitations

This systematic review aimed to determine the effectiveness of robot-assisted CD in managing ONFH. The results suggest that robot-assisted CD holds promise in offering a better treatment approach when compared to freehand CD. However, there are several limitations to consider. Robot-assisted CD, a relatively newer procedure, lacks high-quality randomized controlled trials to validate its efficacy. The included studies were cohort reviews, and the overall evidence grade from these studies is relatively low. Second, the meta-analysis considered only seven studies, pointing to potential search deficiencies and limited sample size. Non-Chinese and English articles were not included, which might have further biased our study. Third, Different levels of familiarity with robotic navigation techniques among operators could introduce significant heterogeneity in some of the data and possible bias in the study results. Finally, the included studies were conducted in China, which may affect the reliability of the conclusions. However, we still hope that more physicians will participate in this study.

### Future directions

To better understand robot-assisted CD’s efficacy, addressing these limitations in future research is crucial. Given the surge in robot-assisted technology in medicine, it would also be beneficial to continuously offer training opportunities to budding physicians. More holistic future research could focus on patient quality of life, fracture risk, and patient satisfaction to ensure more reliable outcomes. Meanwhile, with the development of computer technology, artificial intelligence, and mechanical manufacturing, robot-assisted technology will continue to evolve to improve the safety and effectiveness of surgery. Although more relevant, high-quality research is lacking, there is clinical value in advancing continued research in robot-assisted technology.

## Conclusion

We conclude that there is inadequate evidence to regularly recommend robot-assisted therapy for the treatment of patients with ONFH. In future studies, higher-quality research will be needed to better define robot-assisted therapy as a treatment option for ONFH.

### Electronic supplementary material

Below is the link to the electronic supplementary material.

Supplementary Material 1

Supplementary Material 2

## Data Availability

All data generated or analysed during this study are included in this published article [and its supplementary information files].

## Abbreviations

CD: Core decompression
CI: Confidence interval
GRADE: Grades of Recommendation Assessment Development and Evaluation
HHS: Harris hip score
MD: Mean difference
NOS: Newcastle-Ottawa Scale
ONFH: Osteonecrosis of the femoral head
RR: Risk ratio
VAS: Visual analog scale
