# Supplementary material for: Comparison of clinical efficacy of robot-assisted and freehand core decompression in the treatment of osteonecrosis of the femoral head: a systematic review and meta-analysis
Source: BMC Musculoskelet Disord. 2024 Jun 18;25:476. doi: 10.1186/s12891-024-07592-x (PMC11184764; doi:10.1186/s12891-024-07592-x)
Supplement: Supplementary file 2 — Supplementary Material 2 [file 12891_2024_7592_MOESM2_ESM.docx]

**Supplementary Material**

Comparison of clinical efficacy of robot-assisted and freehand core decompression in the treatment of osteonecrosis of the femoral head: a systematic review and meta-analysis

Contents

Search strategies 2

Table S1. Bias risk assessment results of included retrospect studies. 4

Table S2. Seneitivity analysis for operative duration. 5

Table S3. Seneitivity analysis for intraoperative blood loss volume. 6

Table S4. Seneitivity analysis for frequency of intraoperative fluoroscopies. 7

Table S5. Seneitivity analysis for ΔHHS. 8

Table S6. GRADE evaluation of evidence quality. 9

**Search strategies**

*PubMed:*

#1 ((robotic [Title/Abstract]) OR (robot positioning [Title/Abstract]) OR (navigated [Title/Abstract])

#2 ("osteonecrosis of the femoral head" [Mesh]) OR ((femur head necrosis [Title/Abstract])) OR (ONFH [Title/Abstract])) OR (FHN [Title/Abstract])

#3 #1 AND #2

*Web of Science:*

#1 TS=(robotic OR robot positioning OR navigated)

#2 TS=(osteonecrosis of the femoral head OR femur head necrosis OR ONFH OR FHN)

#3 #1 AND #2

*Embase:*

#1 'robotic'/exp

#2 'robot positioning':ab,ti OR 'navigated':ab,ti

#3 #1 OR #2

#4 'osteonecrosis of the femoral head'/exp

#5 'femur head necrosis':ab,ti OR 'ONFH':ab,ti OR 'FHN':ab,ti

#6 #4 OR #5

#7 #3 AND #6

*Cochrane Central Register of Controlled Trials：*

#1 (robotic):ti,ab,kw OR (robot positioning):ti,ab,kw OR (navigated):ti,ab,kw

#2 MeSH descriptor: [osteonecrosis of the femoral head] explode all trees

#3 (femur head necrosis):ti,ab,kw OR (ONFH):ti,ab,kw OR (FHN):ti,ab,kw

#4 #2 OR #3

#5 #1 AND #4

*Chinese National Knowledge Infrastructure (CNKI)：*
( ( ( 主题=机器人 或者 题名=机器人 或者 v_subject=中英文扩展(机器人) 或者 title=中英文扩展(机器人)) 或者 (主题=机器人定位 或者 题名=机器人定位 或者 v_subject=中英文扩展(机器人定位) 或者 title=中英文扩展(机器人定位)) ) 或者 ( (主题=导航 或者 题名=导航 或者 v_subject=中英文扩展(导航) 或者 title=中英文扩展(导航)) ) 并且 ( (主题=股骨头坏死 或者 题名=股骨头坏死 或者 v_subject=中英文扩展(股骨头坏死) 或者 title=中英文扩展(股骨头坏死)) 或者 (主题=股骨头缺血性坏死 或者 题名=股骨头缺血性坏死 或者 v_subject=中英文扩展(股骨头缺血性坏死) 或者 title=中英文扩展(股骨头缺血性坏死)) )

*Chinese Science and Technology Periodical database：*

(题名或关键词=机器人 OR 题名或关键词=导航) AND (题名或关键词=股骨头坏死 OR 题名或关键词=股骨头缺血性坏死)

*WanFang database：*

(主题:(机器人) or 主题:(导航) and (主题:(股骨头坏死) or 主题:(股骨头缺血性坏死)

*Chinese Biological Medicine database：*

#1 "机器人"[不加权:扩展]

#2 ("机器人定位"[常用字段:智能] OR "导航"[常用字段:智能]

#3 ("股骨头坏死"[常用字段:智能] OR "股骨头缺血性坏死"[常用字段:智能])

#4 ((#1) OR (#2))

#5 ((#3) AND ((#4)

**Table S1**

Bias risk assessment results of included retrospect studies.

| Study | Selection | | | | Comparability | Outcome | | | Newcastle-Ottawa Scale |
| --- | --- | --- | --- | --- | --- | --- | --- | --- | --- |
|  | Q1 | Q2 | Q3 | Q4 | Q5 | Q6 | Q7 | Q8 | Overall score |
| Tian et al.,  2023 [22] | 1 | 1 | 1 | 1 | 1 | 1 | 0 | 1 | 7 |
| Liu et al.,  2022 [23] | 1 | 1 | 1 | 1 | 2 | 1 | 0 | 1 | 8 |
| Zhang et al.,  2022 [24] | 1 | 1 | 1 | 1 | 2 | 1 | 0 | 1 | 8 |
| Li et al.,  2022 [25] | 1 | 1 | 1 | 1 | 2 | 1 | 0 | 1 | 8 |
| Luo et al.,  2020 [26] | 1 | 1 | 1 | 1 | 1 | 1 | 0 | 1 | 7 |
| Luo J et al.,  2020 [27] | 1 | 1 | 1 | 1 | 1 | 1 | 0 | 1 | 7 |
| Bi et al.,  2019 [28] | 1 | 1 | 1 | 1 | 2 | 1 | 0 | 1 | 8 |

Notes: Q1. Representativeness of the exposed group; Q2. Representativeness of the non-exposed group; Q3. Identification of exposure factors; Q4. Confirmation of no outcome indicators to be observed at the beginning of the study; Q5. Comparability on the basis of the design or analysis; Q6. Assessment of outcome indicators; Q7. Adequate follow-up duration; Q8. Completeness of follow‐up between the exposed and unexposed groups.

**Table S2**

Seneitivity analysis for operative duration.

| Eliminated study | Heterogeneity | | Effect Model | MD | 95% CI | P Value |
| --- | --- | --- | --- | --- | --- | --- |
|  | P Value | I^2^ (%) |  |  |  |  |
| None | <0.00001 | 98 | Random | -17.60 | -23.41 to -11.78 | <0.0001 |
| Tian et al.,  2023 [22] | <0.00001 | 96 | Random | -19.57 | -24.39 to -14.74 | <0.00001 |
| Liu et al.,  2022 [23] | <0.00001 | 98 | Random | -15.88 | -21.95 to -9.81 | <0.00001 |
| Zhang et al.,  2022 [24] | <0.00001 | 98 | Random | -17.33 | -25.62 to -9.03 | <0.0001 |
| Li et al.,  2022 [25] | <0.00001 | 97 | Random | -16.83 | -24.25 to -9.42 | <0.00001 |
| Luo et al.,  2020 [26] | <0.00001 | 98 | Random | -18.21 | -24.71 to -11.70 | <0.00001 |
| Luo J et al.,  2020 [27] | <0.00001 | 97 | Random | -19.19 | -24.99 to -13.40 | <0.00001 |
| Bi et al.,  2019 [28] | <0.00001 | 98 | Random | -16.55 | -22.90 to -10.19 | <0.00001 |

**Table S3**

Seneitivity analysis for intraoperative blood loss volume.

| Eliminated study | Heterogeneity | | Effect Model | MD | 95% CI | P Value |
| --- | --- | --- | --- | --- | --- | --- |
|  | P Value | I^2^ (%) |  |  |  |  |
| None | <0.00001 | 97 | Random | -19.98 | -28.84 to -11.11 | <0.0001 |
| Tian et al.,  2023 [22] | <0.00001 | 96 | Random | -23.31 | -31.63 to -14.98 | <0.00001 |
| Liu et al.,  2022 [23] | <0.00001 | 97 | Random | -15.42 | -24.68 to -6.16 | 0.001 |
| Zhang et al.,  2022 [24] | <0.00001 | 98 | Random | -21.53 | -31.08 to -11.97 | <0.0001 |
| Li et al.,  2022 [25] | <0.00001 | 97 | Random | -23.12 | -32.29 to -13.94 | <0.00001 |
| Luo et al.,  2020 [26] | <0.00001 | 97 | Random | -20.14 | -36.95 to -3.32 | 0.02 |
| Luo J et al.,  2020 [27] | <0.00001 | 94 | Random | -17.57 | -29.90 to -5.24 | 0.005 |

**Table S4**

Seneitivity analysis for frequency of intraoperative fluoroscopies.

| Eliminated study | Heterogeneity | | Effect Model | MD | 95% CI | P Value |
| --- | --- | --- | --- | --- | --- | --- |
|  | P Value | I^2^ (%) |  |  |  |  |
| None | <0.00001 | 98 | Random | -6.60 | -9.01 to -4.20 | <0.00001 |
| Tian et al.,  2023 [22] | <0.00001 | 98 | Random | -7.02 | -9.73 to -4.30 | <0.00001 |
| Zhang et al.,  2022 [24] | 0.0007 | 82 | Random | -5.70 | -6.86 to -4.54 | <0.00001 |
| Li et al.,  2022 [25] | <0.00001 | 97 | Random | -6.99 | -9.69 to -4.29 | <0.00001 |
| Luo et al.,  2020 [26] | <0.00001 | 98 | Random | -6.93 | -9.84 to -4.02 | <0.00001 |
| Luo J et al.,  2020 [27] | <0.00001 | 98 | Random | -6.39 | -9.41 to -3.38 | <0.0001 |

**Table S5**

Seneitivity analysis for ΔHHS.

| Eliminated study | Heterogeneity | | Effect Model | MD | 95% CI | P Value |
| --- | --- | --- | --- | --- | --- | --- |
|  | P Value | I^2^ (%) |  |  |  |  |
| None | 0.006 | 67 | Random | 0.51 | -1.34 to 2.35 | 0.59 |
| Tian et al.,  2023 [22] | 0.02 | 62 | Random | 0.07 | -1.85 to 1.99 | 0.94 |
| Liu et al.,  2022 [23] | 0.15 | 39 | Fixed | -0.29 | -1.69 to 1.10 | 0.68 |
| Zhang et al.,  2022 [24] | 0.01 | 65 | Random | 1.04 | -1.07 to 3.16 | 0.33 |
| Li et al.,  2022 [25] | 0.007 | 69 | Random | 0.92 | -1.43 to 3.26 | 0.44 |
| Luo et al.,  2020 [26] | 0.003 | 72 | Random | 0.70 | -1.32 to 2.73 | 0.49 |
| Luo J et al.,  2020 [27] | 0.003 | 72 | Random | 0.49 | -1.62 to 2.59 | 0.65 |
| Bi et al.,  2019 [28] | 0.003 | 72 | Random | 0.68 | -1.43 to 2.79 | 0.53 |

**Table S6**

GRADE evaluation of evidence quality.

| Outcome, number of sties  (number of participants) | Limitation in study design ^1^ | Risk of bias ^2^ | Inconsistency of results ^3^ | Indirectness ^4^ | Imprecision ^5^ | Publication bias ^6^ | Overall quality of evidence |
| --- | --- | --- | --- | --- | --- | --- | --- |
| the operative duration  7 Studies (355) | -1 | 0 | -1 | 0 | -1 | 0 | Very low |
| the intraoperative blood loss volume  6 Studies (335) | -1 | 0 | -1 | 0 | -1 | 0 | Very low |
| the frequency of intraoperative fluoroscopies  5 Studies (304) | -1 | 0 | -1 | 0 | -1 | 0 | Very low |
| the ΔVAS score  5 Studies (252) | -1 | 0 | 0 | 0 | -1 | 0 | Low |

| ^3^ Inconsistency of results.The extent of overlap of confidence intervals and I^2^ test | | | |
| --- | --- | --- | --- |
| Variable | Confidence intervals overlapping | Test of heterogeneity | I^2^ |
| the operative duration | Yes | P < 0.00001 (significant) | 98% (Substantial) |
| the intraoperative blood loss volume | Yes | P < 0.00001 (significant) | 97% (Substantial) |
| the frequency of intraoperative fluoroscopies | Yes | P < 0.00001 (significant) | 98% (Substantial) |
| the ΔVAS score | Yes | P = 0.24(non-significant) | 28% (Moderate) |

| ^6^ Publication bias. Summary of funding according to the outcome variables | | | | |
| --- | --- | --- | --- | --- |
| Variable | Number of studies that reported no funding or no influence | Number of studies that unreported  funding | Number of studies that reported any type of  funding | Total |
| the operative duration | 1 | 2 | 4 | 7 |
| the intraoperative blood loss volume | 0 | 2 | 4 | 6 |
| the frequency of intraoperative fluoroscopies | 0 | 2 | 3 | 5 |
| the ΔVAS score | 1 | 1 | 3 | 5 |

| ^6^ Publication bias. Summary of conflict of interest according to the outcome variables | | | |
| --- | --- | --- | --- |
| Variable | Number of studies declaring no conflict of interest | Number of studies unreported conflict of interest | Total |
| the operative duration | 4 | 3 | 7 |
| the intraoperative blood loss volume | 3 | 3 | 6 |
| the frequency of intraoperative fluoroscopies | 3 | 2 | 5 |
| the ΔVAS score | 3 | 2 | 5 |

Rate down if: ^1^ Observational study.

^2^ > 25 % of the participants from studies with a high risk of bias.

^3^ Heterogeneity was based on the extent of overlap of confidence intervals and I^2^ test (>50 %);

^4^ > 25 % of studies had a poor representation of outcome (when definitions of the outcome varied) or population (non-general population);

^5^ Fewer than 400 participants in the pooling.

^6^ Publication bias was based on the funnel plot and Egger’s test (*Unclear for outcomes with less than 10 studies included), the sample size of the included studies, sponsorship and/or conflict of interest report.
